# The comparison of oxidative stress effect in classic and tubeless percutaneous nephrolithotomy

**DOI:** 10.1007/s11255-018-2003-x

**Published:** 2018-10-09

**Authors:** Piotr Bryniarski, Sławomir Kasperczyk, Paweł Rajwa, Paweł Stelmach, Małgorzata Cisowska-Babraj, Andrzej Paradysz

**Affiliations:** 1 Department of Urology, School of Medicine with the Division of Dentistry in Zabrze, Medical University of Silesia, 3 Maja Street 13-15, 41-800 Zabrze, Poland; 2 Department of Biochemistry, School of Medicine with the Division of Dentistry in Zabrze, Medical University of Silesia, Jordana Street 19, 41-800 Zabrze, Poland

**Keywords:** Kidney stone disease, Urolithiasis, Percutaneous nephrolithotomy, Oxidative stress

## Abstract

**Purpose:**

Surgical stone treatment induces oxidative stress in kidney tissue. We hypothesized that tubeless percutaneous nephrolithotomy (tPCNL) may induce less oxidative stress than classic percutaneous nephrolithotomy (cPCNL) with nephrostomy tube.

**Methods:**

Seventy-two consecutive patients with kidney stones qualified for PCNL were enrolled in the study. Patients were assigned to one of two groups (first group 33 patients—cPCNL and second group 39 patients—tPCNL). Four urine samples were collected in four consecutive days, starting the day before operation. Four oxidative stress markers were analyzed in each sample: catalase (CAT), protein sulfhydryl group (SH), total antioxidant capacity (TAC) and superoxide dismutase (SOD).

**Results:**

Baseline mean levels of CAT (IU/l), SH (μmol/l), TAC (mmol/l) and SOD (NU/ml) were 19.4 versus 11.7; 18 versus 58.7; 2.02 versus 1.99; 20.5 versus 22.6 in cPCNL and tPCNL group, respectively. On day two, the levels were 89 versus 104.9; 334.7 versus 518.9; 1.87 versus 1.79; 33.7 versus 41.4, respectively. On the third day, the levels were: 67.4 versus 28.3; 206.8 versus 306.9; 2.01 versus 2.06; 38.2 versus 36.6, respectively. On the fourth day, the concentrations were 47.4 versus 18.5; 129.3 versus 208.7; 2 versus 2.06; 35 versus 45.2, respectively. Significant differences were observed only for CAT and TAC concentrations in days 3 (*p* = 0.04 and 0.04) and 4 (*p* = 0.02 and < 0.001) in favor of tPCNL.

**Conclusions:**

CAT, SH and SOD significantly rise after operation. TAC represents the inversion of other parameters. CAT is significantly lower, and TAC is significantly higher in tPCNL postoperatively favoring this method.

## Introduction

The pivotal milestone in the minimally invasive treatment of large kidney stones was firstly described by Fernstrom and Johansson, who performed stone removal procedures using percutaneous access [1]. Percutaneous nephrolithotomy (PCNL) should be performed, according to the European Association of Urology (EUA) and American Urological Association (AUA) guidelines, in patients with renal calculi over 2 cm in diameter, irrespective of the location, and may also be utilized for lower pole stones over 1.5 cm [2–4]. In the classic percutaneous nephrolithotomy technique (cPCNL), the nephrostomy tube is used to allow access for the second-look procedure, to secure urinary drainage in the case of ureteral obstruction and to compress kidney parenchyma to stop bleeding from the surgical site [2, 5–7]. In 1997, Bellman introduced the tubeless percutaneous nephrolithotomy (tPCNL) method, which is currently gaining more and more recognition among urologists [2, 8]. It seems that the advantage of tPCNL over cPCNL is associated primarily with a shorter hospitalization time, less pain and a significant reduction in the use of analgesics [5]. PCNL leads to an increase in oxidative stress marker (OSM) concentrations (activity), which reflects a certain imbalance between released free radicals from tissues and the antioxidant capacity of the environment [9–11]. Our primary goal was to compare cPCNL and tPCNL in terms of OSMs concentrations. The secondary aim was to find any factors that might influence OSMs after PCNL.

## Patients and methods

### Inclusion and exclusion criteria

The study was performed in accordance with the ethical standards laid down in the 1964 Declaration of Helsinki. Informed consent was obtained from all individual participants included in the study. The study was conducted between September 2016 and May 2018. Seventy-two patients who underwent PCNL procedure at our department were enrolled in the study. The first group consisted of 33 patients where cPCNL with nephrostomy tube after operation was utilized. The second group comprised 39 patients where tubeless PCNL was applied.

Inclusion criteria consisted of:

- Patients with stone over 2 cm in diameter in a kidney
- Patients with stones 1–2 cm who wished to have PCNL instead of retrograde intrarenal surgery or shockwave lithotripsy (SWL)
- Patients with stones 1–2 cm with contraindications for SWL

Exclusion criteria consisted of:

- Residual stones after surgery and need for a second look
- Double-J stent or nephrostomy catheter inserted preoperatively
- Persistent bacteriuria despite antibiotic therapy before operation
- Bleeding diathesis
- Solitary kidney
- Ureteral obstruction
- Prolonged procedure (more than 3 h)
- Intraoperative difficulties or complications
- Severe bleeding

### Classic and tubeless PCNL

All patients qualified for PCNL had contrast-enhanced computed tomography performed before surgery. All patients are admitted to hospital 2 days before surgery for additional consultations, imaging (ultrasound and X-ray), blood and urine tests. Two kinds of PCNL were utilized: classic (with nephrostomy tube)—the first group, and tubeless—the second group. Briefly, we operate patients in prone position with utilization of Amplatz dilators and sheaths. Patients are operated in general anesthesia. Perioperatively second-generation cephalosporin is used as antibiotic prophylaxis. Puncture and tract formation are done by urologist under fluoroscopic guidance. We use 26 Fr nephroscope with ultrasound as well as pneumatic lithotripter to disintegrate the stone. At the end of PCNL, either reentry Malecot (16Fr) nephrostomy or TachoSil® was inserted into kidney [12–14]. TachoSil® is a kind of a patch and has two adherent layers. The outer layer is composed of fibrinogen and thrombin that promote coagulation process. The inner layer has a honeycomb structure and is composed of equine collagen. Platelets with coagulation factors form clot in the yellow layer that is surrounded and attached to kidney parenchyma on one side and equine collagen on the other side. Such mechanism of action ensures good hemostasis [12]. There were no patients operated with totally tubeless technique in this study. Ureteral catheter (in tPCNL) was removed next day in the morning after operation. Reentry Malecot catheter was removed 2 days after operation. There was no formal randomization. Two senior urologists performed PCNL procedures in this study. The first one (P.B.) performed only tubeless, while the second one (A.P.) only classic procedures.

### Measures

Blood and urine measures were taken perioperatively in each patient as in every PCNL procedure performed in our department. In addition, four samples (1 ml) of voided urine were collected every 24 h in the evening. The first sample was taken the day before operation. Samples were immediately frozen and stored in containers. Catalase (CAT) activity in urine was measured by method of Johansson and Borg [15] using an automated analyzer PerkinElmer and expressed in IU/l urine [15]. The urine protein sulfhydryl groups (SH) concentration was determined as described by Koster et al. [16] using an automated PerkinElmer analyzer and expressed in µmol/l [16]. Total antioxidant capacity (TAC) in urine was measured according to Erel [17] using an automated PerkinElmer analyzer and expressed in mmol/l [17]. Determination of superoxide dismutase (SOD) activity was done by the method of Oyanagui [18]. The activity of SOD is equal to one nitric unit (NU) when it inhibits nitric ion production by 50%. Activity of SOD was expressed in NU/ml of urine [18].

### Statistical analysis

The distributions of oxidative stress parameters were not normal; thus, logarithmic transformation of data was applied. However, for better visualization, means of absolute values were shown in the graphs. General linear model with analysis of covariance with repeated measures and post hoc analysis with Tukey test was utilized to seek for any differences in OSM, hemoglobin and protein concentration in urine in respective days. For other continuous variables without normal distributions, Mann–Whitney *U* test was used. For categorical variables, Chi-square test was applied. Nonparametric correlations with Spearman test were applied to seek for any relation between dependant and independent and within dependant variables. Blood parameters with normal distribution were analyzed with *t* tests. Repeated measures with normal distribution were analyzed with Hotelling’s test. A *p* values less than 0.05 were considered significant. Statistical analysis was conducted using Statistica Statsoft™ version 13.1.

## Results

Preoperative characteristics of patients are given in Table 1. As shown, there were no apparent differences between groups. In order to search for any relationship between continuous preoperative variables (like sodium, potassium, creatinine concentration) and OSM concentrations, we performed correlation tests, but there was no significance in any of them. Similarly, in order to exclude any bias, we compared preoperative continuous variables between categorical variables (i.e., sex, infracostal/intercostals access, uni-/multitract access, accessed calyx) but we also did not find any significance. Postoperative characteristics of our groups are given in Table 2. Only hospitalization time was favorable for tPCNL. Table 3 shows the comparison of pre- and postoperative characteristics of analyzed groups.

**Table 1:** Demographic and preoperative clinical characteristics of analyzed groups

	Classic PCNL (*n* = 33)	Tubeless PCNL (*n* = 39)	*P* value
Age, year, median	58	53	0.56
Sex, no. (%)			
Female	20 (27.7)	16 (22.2)	0.1
Male	13 (18)	23 (31.9)	
BMI, kg/m^2^, mean (SD)	29 (6)	29.2 (4.8)	0.8
Hypertension, *n* (%)			
Yes	18 (25)	18 (25)	0.47
No	15 (20.8)	21 (29.1)	
Diabetes, *n* (%)			
Yes	7 (9.7)	3 (4.17)	0.18
No	26 (36.1)	36 (50)	
Sodium concentration, mmol/l, mean (SD)	142.6 (2.3)	141.9 (2.3)	0.2
Potassium concentration, mmol/l, mean (SD)	4.4 (0.3)	4.3 (0.3)	0.3
Creatinine concentration, µmol/l, mean (SD)	81 (20.5)	80.4 (17.4)	0.9
Hemoglobin, g/dl, mean (SD)	14.5 (1.3)	14.8 (1.6)	0.29
White blood cells, n/mcl, mean (SD)	8.6 (2.2)	7.9 (1.9)	0.18
Platelet count, n/mcl, mean (SD)	248.1 (49.1)	256.4 (79.4)	0.6
Leukocyturia > 5 per high power field, *n* (%)			
Yes	20 (27.7)	17 (23.6)	0.15
No	13 (18)	22 (30.5)	
Side, *n* (%)			
Left	19 (26.3)	23 (31.9)	0.9
Right	14 (19.4)	16 (22.2)	
Stone diameter, mm, mean (SD)	28.9 (13.4)	25.7 (12.9)	0.3
Stone position, *n* (%)			
Calyx	7 (9.7)	14 (19.4)	0.24
Pelvis	10 (13.8)	13 (18)	
Staghorn	16 (22.2)	12 (16.6)	

**Table 2:** Postoperative characteristics of analyzed groups

	Classic PCNL (*n* = 33)	Tubeless PCNL (*n* = 39)	*P* value
Operation time, min, mean (SD)	109 (34.8)	100 (34.2)	0.2
Access, *n* (%)			
Infracostal	21 (29.1)	26 (36.1)	0.78
Intercostal	12 (16.6)	13 (18)	
Multitract access, *n* (%)			
Yes	5 (6.9)	3 (4.1)	0.31
No	28 (38.8)	36 (50)	
Accessed calyx, *n* (%)			
Upper	1 (1.3)	0	0.46
Middle	8 (11.1)	11 (15.2)	
Lower	19 (26.3)	25 (34.7)	
Upper + middle + lower	1 (1.3)	1 (1.3)	
Upper + lower	0	1 (1.3)	
Middle + lower	4 (5.5)	1 (1.39)	
Fever > 38.5 °C^a^, *n* (%)			
Yes	4 (5.5)	6 (8.3)	0.68
No	29 (40.2)	33 (45.8)	
Hospitalization time, days, median	7	5	< 0.001

^a^The first day after operation

**Table 3:** Preoperative and postoperative characteristics of analyzed groups

	Preoperatively		*P* value^a^	6 h after surgery		*P* value^a^	*P* value^b^
	cPCNL (*n* = 33)	tPCNL (*n* = 39)		cPCNL (*n* = 33)	tPCNL (*n* = 39)		
Hemoglobin, g/dl, mean (SD)	14.5 (1.37)	14.8 (1.6)	0.29	12.5 (1.52)	13.2 (1.66)	0.07	0.17
White blood cells, n/mcl, mean (SD)	8.6 (2.2)	7.9 (1.9)	0.18	12.4 (5.1)	12 (4.6)	0.72	0.31
Platelet count, n/mcl, mean (SD)	248.1 (49.1)	256.4 (79.4)	0.6	201.8 (53.6)	217.6 (68)	0.2	0.41
Creatinine µmol/l, mean (SD)	81 (20.5)	80.4 (17.4)	0.9	84 (20)	83.9 (18.4)	0.9	0.99
Sodium concentration, mmol/l, mean (SD)	142.6 (2.3)	141.9 (2.3)	0.21	140.3 (3.5)	140.4 (2.92)	0.84	0.4
Potassium concentration, mmol/l, mean (SD)	4.4 (0.3)	4.3 (0.3)	0.3	4.2 (0.5)	4.2 (0.3)	0.7	0.5

^a^ *t* test

^b^Hotelling’s test

OSMs were positively (negatively for TAC) and significantly correlated with each other in respective days. Protein and hemoglobin concentrations in urine were positively and significantly correlated. Similarly CAT, SOD and SH were positively and significantly correlated with hemoglobin and protein concentration in urine. TAC was positively and significantly correlated with protein but not hemoglobin concentration in respective days. Of all OSMs, SOD had the highest correlation coefficient with hemoglobin and protein concentration (*r* = 0.8; *r* = 0.72 with *p* < 0.05 at day 2, respectively).

**Fig. 1:**
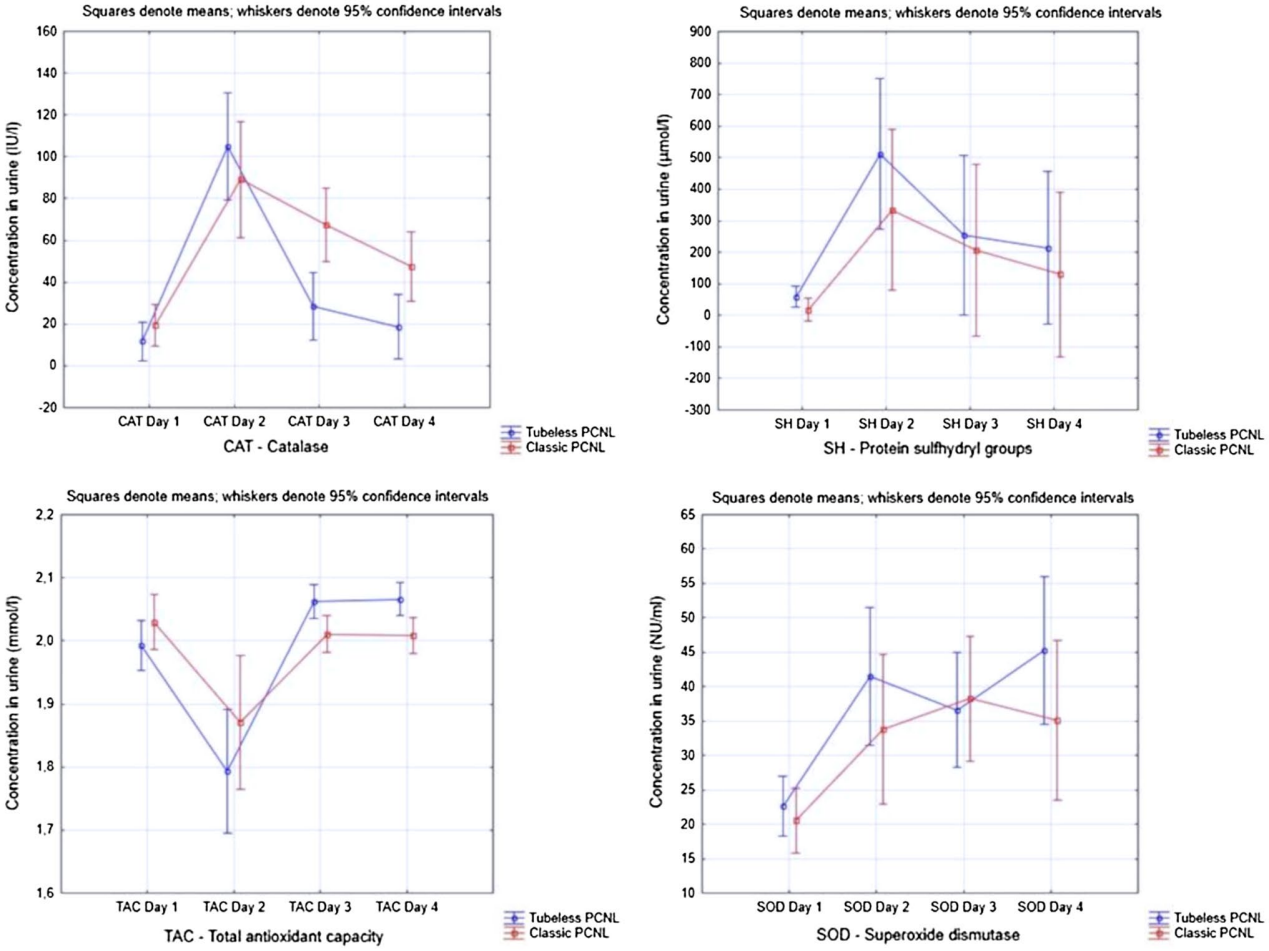
Oxidative stress markers activity in four consecutive days

**Fig. 2:**
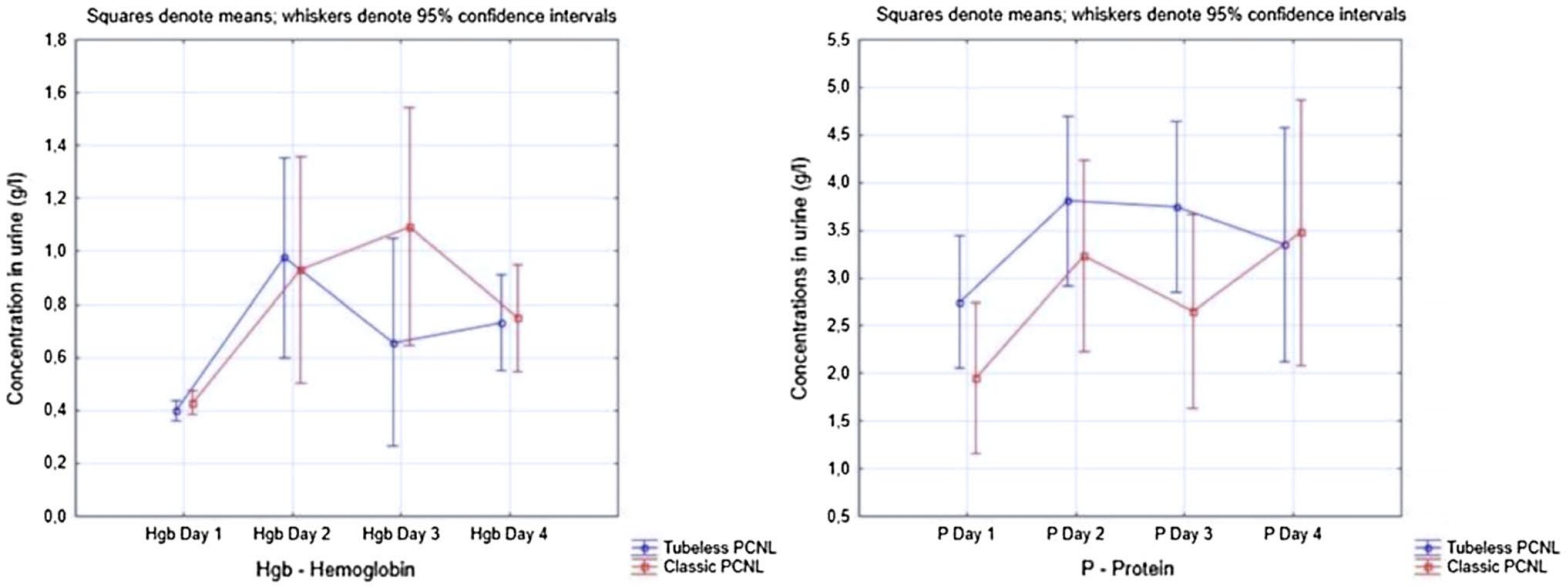
Protein and hemoglobin concentration in four consecutive days

In order to exclude the influence of protein and hemoglobin concentration on OSMs, analysis of covariance was utilized to test the pure oxidative stress effect of operation and nephrostomy tube. The differences in OSM concentrations between cPCNL and tPCNL are given in Table 4. As shown only CAT and TAC concentrations were significantly favorable in tPCNL group in day 3 and 4. Figure 1 represents means of OSM concentrations in respective days. Figure 2 shows hemoglobin and protein concentration in urine in respective days. They all show that surgery itself triggers the highest stress effect at the day of PCNL (Table 5).

**Table 4:** Concentrations of oxidative stress markers in urine

	Classic PCNL (*n* = 33)	Tubeless PCNL (*n* = 39)	*P* value
Day 1			
Protein, g/l, mean (SD)	1.9 (1.3)	2.4 (1.5)	0.24
Hemoglobin, g/l, mean (SD)	0.3 (0.1)	0.3 (0.1)	0.48
CAT, IU/l, mean (SD)	19.4 (41.4)	11.7 (9.6)	0.86
SH, µmol/l, mean (SD)	18 (52.6)	58.7 (133.7)	0.08
TAC, mmol/l, mean (SD)	2.02 (0.08)	1.99 (0.1)	0.1
SOD, NU/ml, mean (SD)	20.5 (12.3)	22.6 (14.5)	0.75
Day 2			
Protein, g/l, mean (SD)	2.5 (1.8)	3.1 (2.2)	0.42
Hemoglobin, g/l, mean (SD)	0.7 (0.7)	0.7 (0.9)	0.7
CAT, IU/l, mean (SD)	89 (73.2)	104.9 (84.6)	0.42
SH, µmol/l, mean (SD)	334.7 (459.2)	518.9 (899.4)	0.73
TAC, mmol/l, mean (SD)	1.87 (0.2)	1.79 (0.3)	0.49
SOD, NU/ml, mean (SD)	33.7 (25.1)	41.4 (35.8)	0.49
Day 3			
Protein, g/l, mean (SD)	2.4 (1.8)	3.1 (2)	0.09
Hemoglobin, g/l, mean (SD)	0.9 (1.3)	0.5 (0.3)	0.49
CAT, IU/l, mean (SD)	67.4 (68.6)	28.3 (26.5)	0.04
SH, µmol/l, mean (SD)	206.8 (473.1)	306.9 (1018.7)	0.91
TAC, mmol/l, mean (SD)	2.01 (0.09)	2.06 (0.07)	0.04
SOD, NU/ml, mean (SD)	38.2 (26.1)	36.6 (26.3)	0.75
Day 4			
Protein, g/l, mean (SD)	2.9 (2.7)	2.9 (2.5)	0.59
Hemoglobin, g/l, mean (SD)	0.6 (0.6)	0.5 (0.4)	0.87
CAT, IU/l, mean (SD)	47.4 (67.6)	18.5 (21.7)	0.02
SH, µmol/l, mean (SD)	129.3 (428.7)	208.7 (929.1)	0.33
TAC, mmol/l, mean (SD)	2.00 (0.1)	2.06 (0.03)	< 0.001
SOD, NU/ml, mean (SD)	35 (30.3)	45.2 (36)	0.16

**Table 5:** Post hoc tests for repeated measures in analyzed groups

	Classic PCNL (*n* = 33)				Tubeless PCNL (*n* = 39)			
	CAT 1	CAT 2	CAT 3	CAT 4	CAT 1	CAT 2	CAT 3	CAT 4
CAT 1		**0.000032**	**0.000136**	0.124724		**0.000032**	0.666725	0.996615
CAT 2	**0.000032**		0.429027	**0.001685**	**0.000032**		**0.000032**	**0.000032**
CAT 3	**0.000136**	0.429027		0.540484	0.666725	**0.000032**		0.972063
CAT 4	0.124724	**0.001685**	0.540484		0.996615	**0.000032**	0.972063	

	SH 1	SH 2	SH 3	SH 4	SH 1	SH 2	SH 3	SH 4
SH 1		**0.031142**	0.905462	0.995153		**0.021457**	0.849918	0.950017
SH 2	**0.031142**		0.988707	0.859574	**0.021457**		0.560200	0.369050
SH 3	0.905462	0.988707		0.999529	0.849918	0.560200		0.999993
SH 4	0.995153	0.859574	0.999529		0.950017	0.369050	0.999993	

	TAC 1	TAC 2	TAC 3	TAC 4	TAC 1	TAC 2	TAC 3	TAC 4
TAC 1		**0.004384**	0.999852	0.999665		**0.000038**	0.632244	0.562615
TAC 2	**0.004384**		**0.021110**	**0.025466**	**0.000038**		**0.000032**	**0.000032**
TAC 3	0.999852	**0.021110**		1.000000	0.632244	**0.000032**		1.000000
TAC 4	0.999665	**0.025466**	1.000000		0.562615	**0.000032**	1.000000	

	SOD 1	SOD 2	SOD 3	SOD 4	SOD 1	SOD 2	SOD 3	SOD 4
SOD 1		**0.024707**	**0.000365**	**0.008217**		**0.000042**	**0.004369**	**0.000032**
SOD 2	**0.024707**		0.956728	0.999983	**0.000042**		0.897510	0.972115
SOD 3	**0.000365**	0.956728		0.994151	**0.004369**	0.897510		0.281848
SOD 4	**0.008217**	0.999983	0.994151		**0.000032**	0.972115	0.281848	

Significant differences are marked bold. CAT 1–4—catalase in days 1–4; SH 1–4—protein sulfhydryl groups in days 1–4; TAC 1–4—total antioxidant capacity in days 1–4; SOD 1–4—superoxide dismutase in days 1–4

## Discussion

OSM concentrations were shown to be affected by various renal conditions including acute renal injury, urolithiasis, shockwave lithotripsy, PCNL [9, 19, 20]. Iodinated contrast media were also shown to impact OSMs [21]. Other conditions like renal colic do not increase oxidative stress [22]. OSMs represent the extent of oxidative stress and may be used to compare different methods of treatment at subclinical level. They can be measured in either spontaneous urine collection or 24-h urine collection [19]. Clinical usefulness of tPCNL is well documented with less pain, lower usage of pain medications and shorter hospitalization time [23–26]. However, whether tPCNL is favorable on pathophysiological level was, so far, not examined. To study this issue, we decided to measure OSM levels in urine after cPCNL and tPCNL in four consecutive days. There are many OSMs, but among most studied are CAT, total antioxidant capacity, protein sulfhydryl groups and SOD. Interestingly, only CAT and TAC significantly differed at days 3 and 4 between groups, while other two OSM were not. This fact may result from the presence of other pathological states like hiperuricosuria, local inflammation, diet, smoking habit, emotional status, which can affect one of OSMs but do not the others; however, exact cause is not known [19, 27–29]. Unfortunately, due to abundance of such pathological states we were unable to control for all of them in multivariate analysis. Such analysis would require significantly more participants.

To be sure that operation itself would not influence the levels of OSM in days 3 and 4, we adopted strict exclusion criteria. Patients who required nephrostomy tube postoperatively (e.g., severe bleeding, any doubt about completeness of procedure, prolonged procedure) were excluded from the study. If such a bias would occur, it would be visible in OSM concentrations in day 2 (the day of operation). None of the OSM, hemoglobin or protein concentration in urine differed between groups on day 2.

Having the data about protein concentration and hemoglobin concentration in each urine sample, we were able to study any relationship between various groups including tubeless/classic, uni-/multitract, inter-/infracostal access but we were unable to find any significant differences. All OSM showed concentration peak (drop for TAC) at the day of operation and slow normalization in the following days. While SH and SOD were similar between tPCNL and cPCNL in days 3 and 4, CAT and TAC concentrations showed significant difference in favor of tPCNL. In other studies, we confirmed significant relationship between time of operation and oxidative stress but only for SOD concentration (day 2) [9]. Such correlation was significant but weak (*r* = 0.29; *p* < 0.05). This may also result from the fact that SOD mostly reflects amount of blood and protein in urine. Other OSMs were not significantly correlated with operation time. There are some limitations of our study that have to be mentioned. Firstly, there are no other studies regarding the sole effect of nephrostomy tube on OSM in urine. Based on our study, we know that oxidative stress is the highest just after operation and is the result of the trauma to the kidney. In the following days, oxidative stress diminishes but nephrostomy tube keeps it higher in comparison with tubeless technique (at least based on TAC and CAT activity). Secondly, this study was conducted in single tertiary care center. Future studies should include more participants from many urological departments. Thirdly, future studies should include reference (nephrostomy only) group to fully assess oxidative stress after percutaneous surgery of the kidney.

## Conclusions

CAT showed lower and TAC showed higher concentrations after tPCNL favoring this method. Other OSM showed equivalence of both methods. Operation itself triggers the highest oxidative stress which normalize in the following days. SOD concentration in urine is highly dependent on the amount of blood and protein in samples. SOD is also significantly and positively correlated with operation time as the sole OSM. Other factors (like infra/intercostal access, accessed calyx, stone position and diameter) did not influence OSM. The best way to alleviate oxidative stress postoperatively is to perform tPCNL or maintain nephrostomy tube for as short as possible.

## References

[1] Fernstrom I, Johansson B (1976). Percutaneous pyelolithotomy: a new extraction technique. Scand J Urol Nephrol.

[2] Turk C, Petrik A, Sarica K (2016). EAU guidelines on interventional treatment for urolithiasis. Eur Urol.

[3] Assimos D, Krambeck A, Miller NL (2016). Surgical management of stones: American Urological Association/Endourological Society Guideline, part I. J Urol.

[4] Assimos D, Krambeck A, Miller NL (2016). Surgical management of stones: American Urological Association/Endourological Society Guideline, part II. J Urol.

[5] Xun Y, Wang Q, Hu H (2017). Tubeless versus standard percutaneous nephrolithotomy: an update meta-analysis. BMC Urol.

[6] Tirtayasa PMW, Yuri P, Birowo P, Rasyid N (2017). Safety of tubeless or totally tubeless drainage and nephrostomy tube as a drainage following percutaneous nephrolithotomy: a comprehensive review. Asian J Surg.

[7] Knudsen BE (2009). Second-look nephroscopy after percutaneous nephrolithotomy. Ther Adv Urol.

[8] Bellman GC, Davidoff R, Candela J, Gerspach J, Kurtz S, Stout L (1997). Tubeless percutaneous renal surgery. J Urol.

[9] Söylemez H, Bozkurt Y, Penbegül N (2013). Time-dependent oxidative stress effects of percutaneous nephrolithotomy. Urolithiasis.

[10] Huang H-S, Ma M-C (2015). High sodium-induced oxidative stress and poor anticrystallization defense aggravate calcium oxalate crystal formation in rat hyperoxaluric kidneys. PLoS ONE.

[11] Ma M-C, Chen Y-S, Huang H-S (2014). Erythrocyte oxidative stress in patients with calcium oxalate stones correlates with stone size and renal tubular damage. Urology.

[12] Bryniarski P, Bogacki R, Muskała B, Taborowski P, Paradysz A (2016). Tubeless percutaneous nephrolithotomy. Videourology.

[13] Cormio L, Perrone A, Di Fino G (2012). TachoSil® sealed tubeless percutaneous nephrolithotomy to reduce urine leakage and bleeding: outcome of a randomized controlled study. J Urol.

[14] Liatsikos EN, Hom D, Dinlenc CZ (2002). Tail stent versus re-entry tube: a randomized comparison after percutaneous stone extraction. Urology.

[15] Johansson LH, Borg LA (1988). A spectrophotometric method for determination of catalase activity in small tissue samples. Anal Biochem.

[16] Koster JF, Biemond P, Swaak AJ (1986). Intracellular and extracellular sulphydryl levels in rheumatoid arthritis. Ann Rheum Dis.

[17] Erel O (2004). A novel automated direct measurement method for total antioxidant capacity using a new generation, more stable ABTS radical cation. Clin Biochem.

[18] Oyanagui Y (1984). Reevaluation of assay methods and establishment of kit for superoxide dismutase activity. Anal Biochem.

[19] Peluso I, Raguzzini A (2016). Salivary and urinary total antioxidant capacity as biomarkers of oxidative stress in humans. Pathol Res Int.

[20] Thamilselvan S, Khan SR (1998). Oxalate and calcium oxalate crystals are injurious to renal epithelial cells: results of in vivo and in vitro studies. J Nephrol.

[21] Sane AS, Upadhyay AR, Mishra VV, Trivedi HL (2000). Iodinated contrast media induced oxidative stress status in patients undergoing urography. Panminerva Med.

[22] Becel S, Icme F, Celik GG (2015). Evaluation of oxidative stress tests in patients diagnosed with renal colic in the emergency department of Ankara Ataturk Training and Research Hospital, Turkey. J Pak Med Assoc.

[23] Shah H, Khandkar A, Sodha H, Kharodawala S, Hegde S, Bansal M (2009). Tubeless percutaneous nephrolithotomy: 3years of experience with 454 patients. BJU Int.

[24] Shah HN, Kausik VB, Hegde SS, Shah JN, Bansal MB (2005). Tubeless percutaneous nephrolithotomy: a prospective feasibility study and review of previous reports. BJU Int.

[25] Abou-Elela A, Emran A, Mohsen MA, Reyad I, Bedair AS, Kader MA (2007). Safety and efficacy of tubeless percutaneous renal surgery. J Endourol.

[26] Wang J, Zhao C, Zhang C, Fan X, Lin Y, Jiang Q (2012). Tubeless vs standard percutaneous nephrolithotomy: a meta-analysis. BJU Int.

[27] Chien J-W, Wang L-Y, Cheng Y-S, Tsai Y-G, Liu C-S (2014). Urinary 8-hydroxy-2′-deoxyguanosine (8-oxodG) level can predict acute renal damage in young children with urinary tract infection. Biomarkers.

[28] Ciftci H, Verit A, Yeni E, Savas M (2008). Decreased oxidative stress index of urine in patients with urinary tract infection. Urol Int.

[29] Göknar N, Oktem F, Arı E, Doğan Demir A, Torun E (2014). Is oxidative stress related to childhood urolithiasis?. Pediatr Nephrol.

